# Ellagic Acid Inhibits Extracellular Acidity-Induced Invasiveness and Expression of COX1, COX2, Snail, Twist 1, and c-myc in Gastric Carcinoma Cells

**DOI:** 10.3390/nu11123023

**Published:** 2019-12-10

**Authors:** Sung-Chul Lim, Hyoin Hwang, Song Iy Han

**Affiliations:** 1 Department of Pathology, College of Medicine, Chosun University, Gwangju 61452, Korea; 2 BioBank, Chosun University Hospital, Gwangju 61452, Korea; 3 Department of Anatomy, College of Medicine, Chosun University, Gwangju 61452, Korea; 4 Division of Premedical Science, College of Medicine, Chosun University, Gwangju 61452, Korea

**Keywords:** extracellular acidity, ellagic acid, invasion

## Abstract

Extracellular acidity has been implicated in enhanced malignancy and metastatic features in various cancer cells. Gastric cancer cell lines (AGS and SNU601) maintained in an acidic medium have increased motility and invasiveness. In this study, we investigated the effect of ellagic acid, a plant-derived phenolic compound, on the acidity-promoted migration and invasion of gastric cancer cells. Treating cells maintained in acidic medium with ellagic acid inhibited acidity-mediated migration and invasion, and reduced the expression of MMP7 and MMP9. Examining regulatory factors contributing to the acidity-mediated invasiveness, we found that an acidic pH increased the expression of COX1 and COX2; importantly, expression decreased under the ellagic acid treatment. The general COX inhibitor, sulindac, also decreased acidity-mediated invasion and expression of MMP7 and MMP9. In addition, acidity increased the mRNA protein expression of transcription factors snail, twist1, and c-myc; these were also reduced by ellagic acid. Together, these results suggest that ellagic acid suppresses acidity-enhanced migration and invasion of gastric cancer cells via inhibition of the expression of multiple factors (COX1, COX2, snail, twist1, and c-myc); for this reason, it may be an effective agent for cancer treatment under acidosis.

## 1. Introduction

Gastric cancer (GC) is one of the most common cancers. The rate of surgical removal of primary cancer is very high, but a great risk comes from the fact that many patients are diagnosed after the tumor has invaded and spread to other parts of the body. In order to eradicate cancer cells, a high dose of medication is required, which is often accompanied by toxic side effects. Inhibition of metastasis can be a more effective strategy to control the progression of cancer. Extensive studies have been conducted to understand the regulatory mechanisms involved in tumor motility, invasiveness, and metastasis. Recently, metabolic parameters of tumor tissue, such as extracellular acidity, have been suggested to be linked to these events [1,2].

Due to active aerobic and anaerobic glycolysis, solid tumors inevitably form an acidic extracellular environment. Such extracellular acidity is a critical factor driving chemoresistance as well as malignant transformation of tumor cells [3,4]. The role of extracellular acidity in promoting invasiveness and metastasis has been demonstrated in human glioma, melanoma, and prostate cancer cells [4,5,6]. Acidity was shown to modulate the expression of multiple oncogenes and EMT-related transcription factors, such as NFkB, HIF2α, and COX2 [7,8], causing the epithelial properties of tumor cells to switch to a more mesenchymal-like phenotype [9]. A low-pH environment triggers the loss of E-cadherin expression in melanoma cells, and tumor cells primed at acidic pH have an increased adherence to the surface at normal pH in vitro and a higher metastatic potential in vivo [10,11], implicating the role of acidity in the migration of tumor cells from the original acidic tumor tissue to another non-tumor site. Therefore, treatment of tumors exposed to an acidic environment appears to be an important issue. Considerable attempts have been made to overcome adverse effects caused by acidic environment with strategies such as the use of proton pump inhibitors that block release of cellular proton into extracellular spaces, but the results have not been as successful as expected. Thus, it is imperative to find agents that prevent acidity-mediated malignancy, the activity of which is not inhibited by extracellular acidity.

Recently, use of natural medicines has been attracting more attention because of long-established medicinal effects and widely recognized safety. Numerous natural compounds have been extensively examined over the past several decades for their potential in cancer prevention and treatment [12]. Ellagic acid is one of naturally occurring phenolic compounds that has recently received considerable attention due to its diverse pharmacological activities, including antioxidant, anti-inflammatory, and anticancer effects [13]. Ellagic acid is contained in ellagitannins, mainly present in vegetables, nuts, and fruits such as raspberries and pomegranates. Among the various beneficial pharmacological activities of ellagic acid, the capacity to prevent several types of cancers is of particular importance. Ellagic acid and ellagic acid-rich foods have shown preventive and therapeutic effects against multiple types of cancers including colorectal cancer, esophageal cancer, breast cancer, prostate cancer, leukemia, and lymphoma [13,14,15]. The anticancer effect of ellagic acid has shown to be mainly mediated through its antiproliferative and pro-apoptotic actions; however, there are also studies indicating that EA inhibits migration and invasion of prostate cancer cells and bladder cancer cells by inhibiting protease activity or reducing the expression of PD-L1 and VEGFR-2 [16,17].

Here, we examined the potential of ellagic acid as an anti-invasive agent for gastric cancer cells exposed to an acidic environment. We used AGS and SNU601 GC cell lines maintained under acidic pH culture condition and assessed the effect of ellagic acid on their acidity-promoted invasive activity and the mechanisms involved.

## 2. Materials and Methods

### 2.1. Cell Lines and Culture Conditions

SNU-601 and AGS human gastric cancer (GC) cells were obtained from the Korean Cell Line Bank (Seoul, Korea) and American Type Culture Collection (Manassas, VA, USA), respectively. Cells were cultured in RPMI 1640 medium (Invitrogen, Carlsbad, CA, USA) supplemented with 10% (*v*/*v*) fetal bovine serum and 1% PS at 37 °C in an atmosphere containing 5% CO_2_. Drugs were purchased from Calbiochem (San Diego, CA, USA). Acidity-conditioned cells were maintained in pH 6.5-adjusted medium for longer than 3 weeks and subcultured at regular intervals.

### 2.2. Invasion and Migration Assay

To assay cell invasiveness, Matrigel-coated transwell chambers (Corning Costar) were used. Equal numbers of cells maintained in pH 7.4 or pH 6.5 were suspended in RPMI of each pH condition containing 1% FBS. Approximately 200 μL of the cell suspension was added to the upper portion of the insert, and medium containing 5% FBS was added to the lower portion of the inset. After 8 h (for AGS) and 18 h (for SNU601) of incubation at 37 °C in 5% CO_2_, noninvasive cells were removed from the upper surface of the transwell membrane with a cotton swab, and the invaded cells on the lower layer surface were fixed in 4% formaldehyde and stained with crystal violet solution. The numbers of invaded cells were counted or imaged under high-power (×200) microscope magnification (Olympus). For the migration assay, cells incubated at pH 7.4 and pH 6.5 were sampled in the same manner as above and grown in 24-well plates in growth medium. After overnight culture, the middle of the cell surface was scraped with a micropipette tip to make a wound of constant width. Debris was washed out with PBS, and the wound closures were monitored and photographed at 6 h (AGS cells) and 18 h (SNU601 cells) under the microscope (× 100, Olympus). Migration distance was calculated applying the software program HMIAS-2000.

### 2.3. Cytotoxicity Assays

The EZ-cytox viability assay was performed following the manufacturer’s protocol. Briefly, cells were plated in wells of a 24-well plate at a density of 5–8 × 10^4^ cells/well, cultured for 24 h, and then incubated in the growth medium with or without ellagic acid for 48 h. The EZ-cytox solution (Daeillab, Korea) was added to the wells and incubated at 37 °C in a CO_2_ incubator for the last 2 h of incubation, and the plates were read using an enzyme-linked immunosorbent assay plate reader at 450 nm. The absorbance of the untreated cells was set as 100%, and cell survival was expressed as a percentage of this value.

### 2.4. Western Blot Analysis

Treated cells were lysed in a whole-cell lysis buffer (50 mM Hepes, 150 mM NaCl, 1% Triton X-100, 5 mM EGTA, protease inhibitor cocktail), and equal amounts of protein extracts were electrophoretically separated using 10%–12% SDS-PAGE and transferred to a nitrocellulose membrane using standard techniques. The proteins were probed using antibodies for COX1 (ab695, abcam), COX2 (sc-376861, Santa Cruz Biotechnology), and α-tubulin (sc-5286) diluted in TBS solution containing 2% skim milk and incubated overnight at 4 °C. Signals were acquired using an Image Station 4000MM image analyzer (Kodak, NY, USA).

### 2.5. Real-Time Reverse Transcription-Polymerase Chain Reaction

Real-time PCR was performed with the Light Cycler 2.0 (Roche) using the Fast Start DNA Master SYBR Green I Kit (Roche). For verification of the correct amplification product, PCR products were analyzed on a 2% agarose gel stained with ethidium bromide. The sequences of the primers were designed as follows: for β-actin, 5′-GACTATGACTTAGTTGCGTTA-3′ and 5′-GCCTTCATACATCTCAAGTTG-3′, for snail, 5′-GGCTCCTTCGTCCTTCT-3′ and 5′-GGCTGAGGTATTCCTTGTT-3′, for twist1, 5′-CGGGAGTCCGCAGTCTTA-3′ and 5′-CTGGTAGAGGAAGTCGATGT-3′, for c-myc, 5′- GCTTTATCTAACTCGCTGTAGTAAT-3′ and 5′- GCTGCTATGGGCAAAGTTTC-3′. Primers of MMP7 (P310408) and MM9 (P323207) were purchased from Bioneer. PCR was conducted at 95 °C for 10 min, followed by 45 cycles of 95 °C for 15 seconds, 60 °C for 5 seconds, and 72 °C for 7 seconds. Melt curve analysis was performed to confirm that a single product was present. Negative controls without template were included in each run. Data were analyzed using Light Cycler software version 4.0 (Roche, Switzerland). The 2^ΔΔCt^ method was used for analysis of relative gene expression.

### 2.6. RNA Interference (RNAi)

For the RNAi experiment, siRNAs of snail, twist1, and c-myc and a scrambled siRNA control were purchased from Bioneer (Daejeon, Korea). Cells were individually transfected with siRNA oligonucleotides using an Amaxa™ Transfection System (Basel, Switzerland) and grown for 48 h in the acidic pH medium.

### 2.7. Statistical Analysis

All numerical data are presented as mean ± SE of three independent experiments. For statistical analysis, student’s *t*-test was used for simple comparisons, and one-way ANOVA with Tukey’s test was used for multiple comparison test. A *p*-value of 0.05 or less was considered statistically significant.

## 3. Results

### 3.1. Acidic Culture Condition Increases Motility and Invasiveness of Gastric Cancer Cells

**Figure 1:**
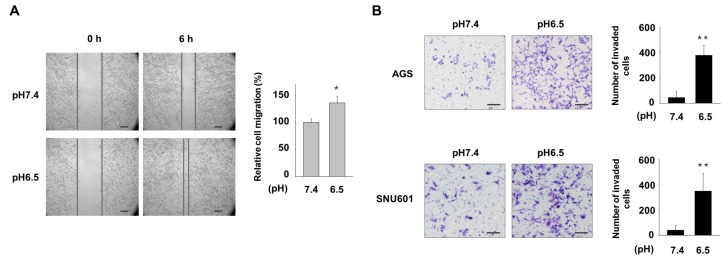
Acidic culture conditions increase migration and invasion of gastric cancer (GC) cells. (**A**) AGS cells were maintained in normal (pH 7.4) or acidic (pH 6.5) medium, and migration of the cells was assessed by using a wound healing assay at 6 h after scratch. (**B**) Invasion of cells cultured in normal or acidic medium was assessed by a matrigel-coated transwell assay. After 6 h for AGS and 18 h for SNU601, invaded cells were detected under a microscope (left) and the number of invaded cells was counted (right). * *p* < 0.05, ** *p* < 0.01 vs. pH 7.4. Scale bar = 100 μm.

Tumor cell migration and invasion are critical events in the process of cancer metastasis. In this study, we first evaluated migratory behavior of AGS gastric cancer cells under normal and acidic pH conditions. Cells maintained in pH 7.4 or pH 6.5-adjusted medium were plated, and motility was detected by wound healing assay. When observed after 6 h, the cells cultured in pH 6.5 medium exhibited higher migration than cells cultured in normal pH medium (Figure 1A). We then assessed the effect of acidic pH condition on invasive ability of AGS and SNU601 cells. The same numbers of cells maintained at pH 7.4 and pH 6.5 were seeded on the inner wells of matrigel-coated transwell plates, and cells infiltrating into downward were detected. Higher numbers of cells cultured at pH 6.5 passed through matrigel than cells cultured at pH 7.4 (Figure 1B). Therefore, acidic conditions may trigger a more migratory and invasive phenotype in gastric cancer cells.

### 3.2. Ellagic Acid Inhibits Acidity-Mediated Migration and Invasion of Gastric Cancer Cells

**Figure 2:**
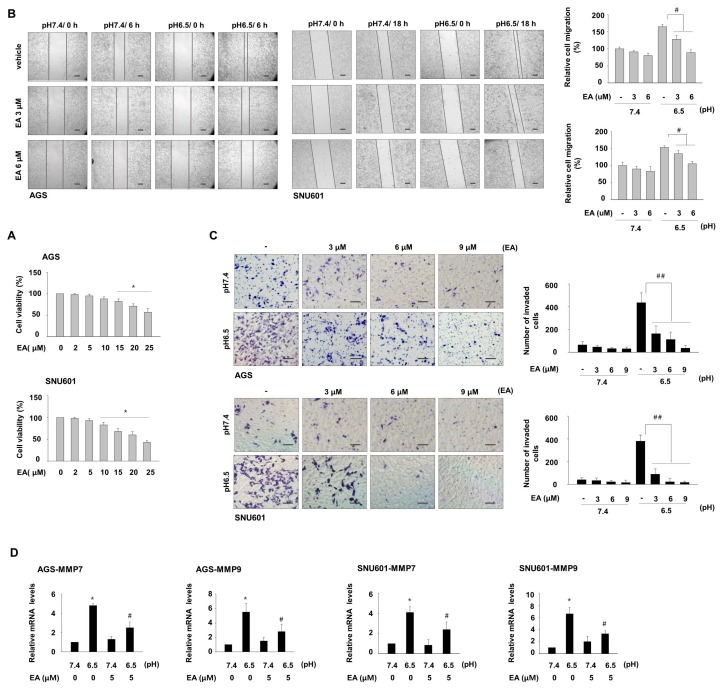
Ellagic acid inhibits acidity-enhanced cell migration and invasion. (**A**) AGS and SNU601 cells were treated with the indicated concentrations of ellagic acid for 48 h, and cell viability was assessed by the EZ-cytox assay. * *p* <0.05 vs. no treatment. (**B**) Cells maintained in normal or acidic medium were further exposed to ellagic acid for 24 h. Then, cell surface was scraped, and migrated cells were detected under microscope (left). Quantitative data are shown (right). (**C**) Cells maintained in normal pH or acidic pH were further incubated at the indicated concentrations of ellagic acid for 24 h; invasion ability was assessed by invasion assay using matrigel-coated transwell system. After 6 h for AGS and 18 h for SNU601, invaded cells were detected under a microscope (left) and the number of invaded cells was counted (right). # *p* < 0.05, ## *p* < 0.01 vs. no ellagic acid at pH 6.5. (**D**) Cells cultured in normal or acidic growth medium were further incubated for 24 h without or with ellagic acid. The cells were then harvested, and mRNA expression of the genes encoding MMP7 and MMP9 was analyzed by real-time PCR. * *p* < 0.05 vs. no treated control at pH 7.4; # *p* < 0.05 vs. no ellagic acid at pH 6.5. Scale bar = 100 μm.

We examined whether ellagic acid affects acidity-promoted migration and invasion of gastric cancer cells. In a cytotoxicity assay, concentrations of ellagic acid greater than 10 μM significantly decreased the viability of these cells (Figure 2A). Thus, concentrations less than 10 μM were used in experiments to specifically study effects on invasiveness, not on cell death. To assess the effect of ellagic acid on acidity-induced migration, cells were pretreated with ellagic acid for 24 h before a scratch in the cell surface was made, and the cells were further incubated in the acidic medium in the presence of ellagic acid. Ellagic acid treatment inhibited wound closure of both cell lines compared with untreated cells (Figure 2B). Furthermore, ellagic acid treatment of cells maintained in acidic medium decreased matrigel infiltration of these cells in a concentration-dependent manner, as detected by the transwell invasion assay. Even at a low concentration (3 μM), ellagic acid treatment reduced the number of invading cells by 66.4% and 78.1%, respectively, in AGS and SNU601 cells compared with untreated cells (Figure 2C). These results suggest that a low concentration of ellagic acid can suppress acidity-promoted invasion of GC cells. We then investigated the expression of regulatory factors involved in migration and invasion and saw that cells cultured under acidic conditions had increased mRNA expression of MMP7 and MMP9 compared with the cells cultured in normal pH medium. Ellagic acid treatment decreased the acidity-induced expression of MMP7 and MMP9, as assessed by real-time PCR (Figure 2D).

### 3.3. EA Decreases Induction of COX1 and COX2, Which Are Involved in Acidity-Promoted GC Invasion

**Figure 3:**
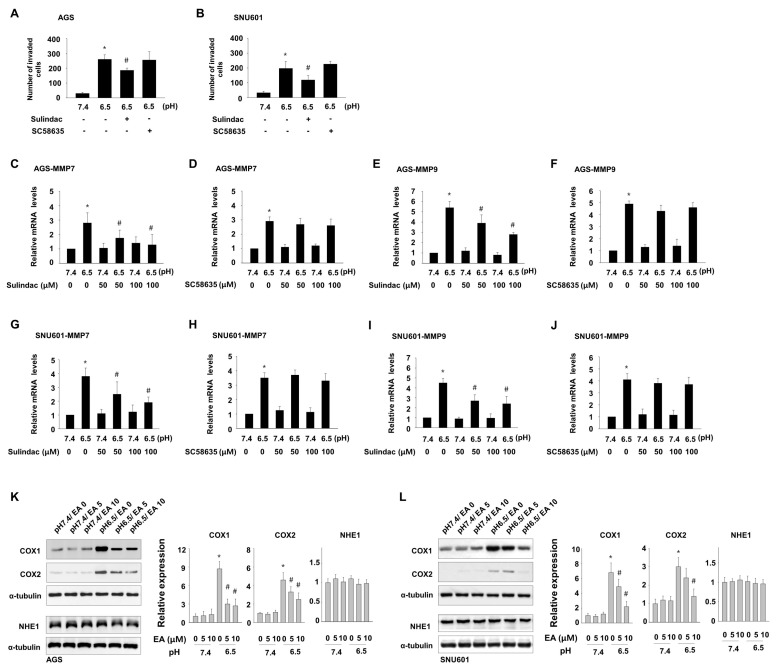
Ellagic acid inhibits expression of COX1 and COX2, which are involved in acidity-enhanced invasion. (**A**,**B**) AGS and SNU601 cells maintained in acidic medium were treated with 100 μM sulindac or 30 μM SC58635 for 24 h. Then, the number of invaded cells was counted. (**C**–**J**) AGS (**C**–**F**) and SNU601 (**G**–**J**) cells maintained in acidic medium were treated with sulindac (**C**, **E**,**G**,**I**) or SC58635 (**D**,**F**,**H**,**J**) for 24 h. The mRNA expression of the genes encoding MMP7 (**C**,**D**,**G**,**H**) and MMP9 (**E**,**F**,**I**,**J**) was subsequently analyzed by real-time PCR. (**K**,**L**) AGS (**K**) and SNU601 (**L**) cells maintained in normal or acidic medium were further incubated for 24 h with or without ellagic acid. Then, total protein extracts were analyzed by immunoblotting with antibodies against COX1, COX2, and NHE1 (left), and densitometry calculations for immunoblotting data are shown (right). * *p* < 0.05 vs. no treated control at pH 7.4; # *p* < 0.05 vs. no ellagic acid at pH 6.5.

To understand the mechanisms by which ellagic acid inhibits acidity-mediated invasiveness in this system, we explored the possibility that the inhibitory effect of ellagic acid is related to COX activity. We detected matrigel invasion ability and mRNA expression of MMP7 and MMP9 of cells grown at low pH in the presence of the general COX inhibitor sulindac, which interferes with both COX1 and COX2 activity, or the specific COX2 inhibitor SC58635. Sulindac significantly suppressed acidity-promoted invasion (Figure 3A,B) and acidity-induced mRNA expression of MMP7 and MMP9 (Figure 3C,E,G,I) in both cell lines. Addition of SC58635 did not affect the number of invading cells (Figure 3A,B) or the levels of MMP7 and MMP9 (Figure 3D,F,H,J). Consistently with this result, exposure of GC cells to acidic medium increased expression of COX1 and COX2, and both levels were reduced by ellagic acid treatment, as detected by immunoblot assay (Figure 3K,L). Therefore, increased expression of COX1 and COX2 seemed to be involved in the acidity-enhanced gastric cancer cell invasion, and the anti-invasive role of ellagic acid may be associated with suppression of COX1 and COX2 induction, although inhibition of COX activity was not sufficient for complete prevention of cell invasion. On the other hand, in endometrial cancer cells, high concentration of ellagic acid was shown to reduce the expression of the Na/H+ exchanger NHE1, a regulator of extracellular acidity often overexpressed in cancer cells [18]. To determine if the inhibitory role of ellagic acid on acidity-induced gastric cancer cell invasion is associated with the regulation of NHE1 expression, we detected the effect of ellagic acid on NHE1 expression; however, low concentration of ellagic acid hardly affected NHE1 protein expression in these cells. Thus, low concentrations of ellagic acid appear to inhibit acidity-induced invasion through other routes including COX pathway.

### 3.4. Ellagic Acid Suppresses Acidity-Mediated Induction of Snail, Twist1, and c-myc

**Figure 4:**
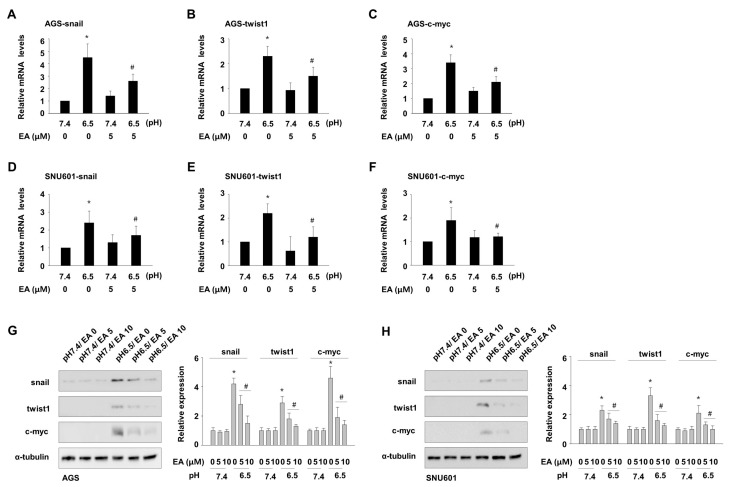
Ellagic acid suppresses acidity-mediated expression of snail, twist1, and c-myc**.** (**A**–**F**) AGS (**A**–**C**) and SNU601 (**D**–**F**) cells cultured in normal or acidic growth medium were further incubated for 24 h without or with 5 μM ellagic acid. Then, mRNA expression of the genes encoding snail (**A**,**D**), twist1 (**B**,**E**), and c-myc (**C**,**F**) was analyzed with real-time PCR. * *p* < 0.05 vs. no treated pH 7.4; # *p* < 0.05 vs. no treated pH 6.5. (**G**,**H**) AGS (**G**) and SNU601 (**H**) cells cultured in normal or acidic growth medium were further incubated for 24 h without or with ellagic acid, then total protein extracts were analyzed by immunoblotting with antibodies against snail, twist1, and c-myc (left). Densitometry calculations for immunoblotting data are shown (right). * *p* < 0.05 vs. no treated control at pH 7.4; # *p* < 0.05 vs. no ellagic acid at pH 6.5.

We observed an increase in the expression of snail, twist1, and c-myc, which are frequently involved in invasion and EMT processes in human cancers. Since COX inhibition alone did not recapitulate the inhibitory effect of ellagic acid, we further investigated the effect of ellagic acid on the acidity-mediated expression of these genes. Real-time PCR showed that addition of ellagic acid decreased the mRNA levels of snail by 37.6% and 26.9% (Figure 4A,D), twist1 by 33.4% and 36.7% (Figure 4B,E), and c-myc by 34.1% and 22.2% (Figure 4C,F), in AGS and SNU601 cells, respectively, as compared with control samples. To confirm the effects of ellagic acid on the increased expression of these factors in acidic culture conditions, immunoblotting analysis was conducted in these cells, as shown in Figure 4G,H. In line with the mRNA expression data, acidic condition elevated protein expression of snail, twist1, and c-myc, and exposure to ellagic acid reduced the expression of these proteins. These results suggest that ellagic acid participates in the inhibition of EMT-inducing factors including snail, twist1, and c-myc under acidic conditions, through which it may contribute to the inhibition of GC cell invasion.

## 4. Discussion

All stages of the progression of tumors and their responses to treatments are greatly influenced by the physical microenvironment surrounding the tumor. Tumor cells must adapt to a wide range of environmental changes within the tumor mass; this is a factor driving transformation of tumor cells to become more resistant or malignant. One of the metabolic parameters of tumor tissue, extracellular acidosis, has been recognized as an important metastatic factor involved in the invasion and metastasis of human glioma, melanoma, and prostate cancer cells [4,5,6] through its influence on the expression of multiple oncogenes and epithelial-mesenchymal transition (EMT)-related genes [7,10]. In line with previous studies on other cell types, our study showed that gastric cancer cells maintained in acidic conditions showed increased motility and invasiveness as well as elevated expression of MMP7 and MMP9, proteolytic enzymes that cleave extracellular matrix proteins, which are well-established biomarkers of invasion and metastasis in human cancers [19,20].

We investigated whether ellagic acid can be a useful agent for metastatic alteration of gastric cancer cells caused by the acidosis. Ellagitanins and their derivatives from black raspberry or pomegranate have shown inhibitory effects on several types of malignancies, including breast, ovarian, and prostate cancer, in studies in vivo and in vitro [16,21,22,23]. The majority of previous studies have focused on the cytotoxic and cytostatic activity of ellagic acid in various types of cancers. For example, ellagic acid was shown to inhibit cell cycle progression by modulating expression of p53, p21, cyclin D1, and cyclin E, and to induce apoptosis by altering the Bax/Bcl-2 ratio in ovarian carcinoma cell lines [24]. Ellagic acid or ellagitanins from pomegranate juice also inhibited proliferation of prostate cancer cells by inhibiting the expression of cyclin D1 and cyclin B1, and triggered the intrinsic and extrinsic apoptosis pathways [25,26,27]. In addition, ellagic acid prevented cell growth and triggered apoptosis in multiple types of cancer cells through inhibition of signal pathways such as PKC, AKT, or PI3K/PKB pathway or induction of the mitochondrial apoptotic pathway [28,29,30,31,32]. Furthermore, ellagic acid also inhibited pancreatic cancer growth in xenografted mice [33].

In our study of gastric cancer cells, high concentrations of ellagic acid also decreased cell viability. However, we focused on the impact of ellagic acid specifically on the invasion capacity of the acidity-exposed cells, since ellagic acid concentrations that affect the survival of cancer cells are relatively high and may cause noncompliance with chemotherapy. The concentrations of ellagic acid used in previous studies ranged from 0.1 to 100 μM, and sensitivity to ellagic acid was highly dependent on cell type. We selected concentrations under which the survival rate of gastric cancer cells was higher than 90% after 48 h incubation. This low dose of ellagic acid reduced acidity-promoted expression of MMP7 and MMP9 and inhibited the migrating and matrigel-infiltrating capability of gastric cancer cells, indicating the inhibitory action of ellagic acid against acidity-enhanced invasiveness. Consistently with our study, the inhibitory roles of ellagic acid in multiple metastatic signaling pathways such as wnt/b-catenin, TGF/smad3, HIF1a, and HIF2a have been also demonstrated in several types of cancer cells such as colon, bladder, and breast cancer cells [17,21,34], although the effect of ellagic acid on malignancy caused by acidity has not been elucidated.

Physiological acidic pH is closely associated with the inflammatory response [35]. Acidity was suggested to be associated with the induction of pro-inflammatory factors, such as TNF-α, IL-1β, and IL-6 [36], and COX2-dependent inflammatory responses [37]. Overexpression of COX2 has been suggested to be related to invasion in several types of cancer cells, including glioma and breast cancer cells [38,39], and inhibition of COX2 was shown to reduce lymphatic metastasis of a human gastric cancer cell line in xenografts [40]. Ellagic acid has been shown to exert anti-inflammatory activity in several disease models and decrease COX2-triggered exacerbation of inflammation [41,42]. Thus, we hypothesized that the inhibitory effect of ellagic acid on acidity-promoted invasion might be mediated through inhibition of COX2. As expected, acidic culture conditions increased COX2 expression, which was decreased by ellagic acid. Interestingly, COX1 expression was also increased by acidic culture and decreased by ellagic acid. A general COX inhibitor but not a selective COX2 inhibitor reduced acidity-induced MMP7 and MMP9 expression and cell invasion. These results suggest that inhibition of COX2 alone may not be sufficient to block MMP7 and MMP9 expression, and additional suppression of COX1 is required to block acidity-mediated invasiveness. In fact, the basal and induced levels of COX1 appeared to be higher than those of COX2. Hence, the inhibitory activity of ellagic acid on both COX1 and COX2 may be associated with the anti-invasive activity in these systems. Although COX1 has long been considered to be constitutively expressed in all tissues and linked to normal physiological functions, not to pathological ones, several recent studies have reported that COX1 can also be upregulated in various pathological conditions and associated with inflammation and cancer [43,44].

Next, we evaluated other genes potentially involved in anti-invasive activity of ellagic acid under acidic conditions. Ellagic acid inhibited extracellular acidity-induced expression of EMT-regulating transcription factors snail and twist1 and proto-oncogene c-myc in our experimental system. EMT is considered a crucial step for cancer metastasis, and expression of snail or twist1 has been shown to be linked to increased mesenchymal marker expression and high metastatic potential in several tumor cells and multiple mouse models. Recent reports have shown an impact of extracellular acidosis on the induction of several EMT-related genes including snail and twist1 [8,9]. Due to loss of E-cadherin in AGS and SNU601 cells, which is a common event in gastric cancer cells, we could not observe acidity-mediated reduction of E-cadherin expression, which is a critical sign of EMT. However, the acidity-induced expression of snail, twist1, and c-myc genes suggests that acidic culture conditions trigger EMT-like gene expression, and thus the ability of ellagic acid to inhibit expression of these genes may be associated with the suppression of an overall shift to a malignant phenotype. However, in contrast with our results, a previous report showed that acidosis lowers c-myc expression in U937 lymphoma cells [45]. c-Myc is a pleiotropic transcription factor that regulates a wide spectrum of cancer cell features, such as tumorigenesis and metastasis [46,47]. The discrepancy may be due to differences in the acid treatment conditions or variance in the cellular context resulting from different cell types. For example, the previous study was performed under acute acidosis, whereas in the current study, cells were exposed to acidic pH medium for a longer period before exposure to ellagic acid because cell viability was significantly reduced in the early stages of acidic condition.

## 5. Conclusions

This study suggests that extracellular acidity plays a role in strongly promoting invasion in gastric cancer cells, and that ellagic acid can be a useful agent in acidic microenvironments for its ability to suppress the expression of multiple malignance-associated genes including COX1, COX2, c-myc, snail, and twist1.
